# A brief, trauma-informed intervention increases safety behavior and reduces HIV risk for drug-involved women who trade sex

**DOI:** 10.1186/s12889-017-4624-x

**Published:** 2017-08-01

**Authors:** Michele R. Decker, Catherine Tomko, Erin Wingo, Anne Sawyer, Sarah Peitzmeier, Nancy Glass, Susan G. Sherman

**Affiliations:** 1 Department of Population, Family & Reproductive Health, Johns Hopkins Bloomberg School of Public Health, Baltimore, MD USA; 2 Women’s Health & Rights Program, Center for Public Health & Human Rights, Johns Hopkins Bloomberg School of Public Health, 615 N. Wolfe Street, E4142, Baltimore, MD 21205 USA; 3 Johns Hopkins School of Nursing, Baltimore, MD USA; 4 Department of Health, Behavior & Society, Johns Hopkins Bloomberg School of Public Health, Baltimore, MD USA; 5 Baltimore City Health Department, Baltimore, MD USA

## Abstract

**Background:**

Female sex workers (FSWs) are an important population for HIV acquisition and transmission. Their risks are shaped by behavioral, sexual network, and structural level factors. Violence is pervasive and associated with HIV risk behavior and infection, yet interventions to address the dual epidemics of violence and HIV among FSWs are limited.

**Methods:**

We used participatory methods to develop a brief, trauma-informed intervention, INSPIRE (Integrating Safety Promotion with HIV Risk Reduction), to improve safety and reduce HIV risk for FSWs. A quasi-experimental, single group pretest-posttest study evaluated intervention feasibility, acceptability and efficacy among FSWs in Baltimore, MD, most of whom were drug-involved (baseline *n* = 60; follow-up *n* = 39 [65%]; non-differential by demographics or outcomes). Qualitative data collected at follow-up contextualizes findings.

**Results:**

Based on community partnership and FSW input, emergent goals included violence-related support, connection with services, and buffering against structural forces that blame FSWs for violence. Qualitative and quantitative results demonstrate feasibility and acceptability. At follow-up, improvements were seen in avoidance of client condom negotiation (*p* = 0.04), and frequency of sex trade under the influence of drugs or alcohol (*p* = 0.04). Women’s safety behavior increased (*p* < 0.001). Participants improved knowledge and use of sexual violence support (*p* < 0.01) and use of intimate partner violence support (*p* < 0.01). By follow-up, most respondents (68.4%) knew at least one program to obtain assistance reporting violence to police. Over the short follow-up period, client violence increased. In reflecting on intervention acceptability, participants emphasized the value of a safe and supportive space to discuss violence.

**Discussion:**

This brief, trauma-informed intervention was feasible and highly acceptable to FSWs. It prompted safety behavior, mitigated sex trade under the influence, and bolstered confidence in condom negotiation. INSPIRE influenced endpoints deemed valuable by community partners, specifically improving connection to support services and building confidence in the face of myths that falsely blame sex workers for violence. Violence persisted; prevention also requires targeting perpetrators, and longer follow-up durations as women acquire safety skills. This pilot study informs scalable interventions that address trauma and its impact on HIV acquisition and care trajectories for FSWs.

**Conclusion:**

Addressing violence in the context of HIV prevention is feasible, acceptable to FSWs, and can improve safety and reduce HIV risk, thus supporting FSW health and human rights.

## Background

Female sex workers (FSWs) bear a disproportionate HIV burden [1]; their risk for acquisition and transmission is shaped by behavioral, sexual network, and structural level factors [2]. Physical and sexual violence victimization by intimate partners, clients, police and other perpetrators is pervasive [3–7]. FSWs’ homicide rate is approximately 17 times higher than the age-standardized rate for women in the general population [8]. Violence enables risk for sexually transmitted infection (STI) including HIV among FSWs [4, 6, 9, 10], and in general populations of women [11]. Drug use intensifies violence-related and HIV-related vulnerability for FSWs [12].

Preventing and responding to violence against FSWs is a global priority to achieve the mutually reinforcing goals of safety and HIV risk reduction [13, 14]. Recommendations emphasize structural goals of legal reform and police accountability, coupled with safety promotion and provision of violence-related health services, legal and psychosocial support at the individual level [13]. Interventions are in their earliest phases. While primary prevention of violence requires changing perpetrator behavior, safety promotion for FSWs is considered a form of harm reduction that lies within women’s control. Safety promotion, e.g., safety tip distribution and sharing “bad date” reports, are widely recommended and standard practice for many FSW programs [13], yet little is known about how these strategies affect behavior. In general populations, disclosing abuse and obtaining support is beneficial [15–17], and can reduce post-traumatic stress [17], self-blame [18], and revictimization [19–21]. Yet for FSWs, accessing justice and violence support services is challenging owing to criminalization, and unique barriers to violence disclosure such as marginalization, self-blame and the myth that transaction trumps consent [7, 22].

To date, integrated, structural approaches show promise in reducing violence and HIV risk for FSWs in India [23]. Tailored, multi-session HIV risk-reduction interventions have been effective in improving safety and reducing some forms of HIV risk [24, 25]. Less is known about how brief interventions that are readily scaleable can impact these outcomes, as well as a wider range of outcomes including safety behavior, knowledge and use of violence-related support services, and attitudes surrounding self-blame and other rape myths specific to sex workers.

The need for interventions is greatest in the urban centers most affected by HIV, where entrenched poverty, substance use, and economic threats create conditions where sex work thrives, and where criminalization and marginalization enable violence against sex workers. Baltimore, MD consistently ranks among the nation’s top 10 major metropolitan areas for HIV diagnoses, with a rate of 22.1 per 100,000 in 2015 [26]. Past research with sex workers in Baltimore confirms significant HIV risk behavior [7, 27], substance use [7, 27], and interest in HIV prophylaxis [28, 29], and the enabling roles of violence and other structural factors [7, 27].

We developed and tested a brief, trauma-informed [30, 31] intervention, INSPIRE (Integrating Safety Promotion with HIV Risk Reduction), to improve safety and reduce HIV risk among FSWs. INSPIRE blends supportive discussion with safety promotion and harm reduction, and support for accessing violence-related services. This approach harnesses outreach workers as a natural conduit for informal, comfortable, open and nonjudgmental discussion.

We describe participatory intervention development, and a quasi-experimental, single group pretest-posttest study with qualitative interviews for context to understand intervention feasibility, acceptability and effect on safety and HIV risk behavior among drug-involved FSWs in Baltimore, MD.

## Methods

### Community-participatory intervention development

INSPIRE was developed, implemented and evaluated via the participatory methods recommended for responding to violence against sex workers [13]. Guiding principles included collaborative problem definition and resolution [32, 33]. Prior preparatory work included extensive in-depth interviews [7] and alliance-building with FSWs, health and social service providers, and city health officials. Coalition participants included service providers from local violence support programs and organizations that work with the sex industry, or whose clients include significant numbers of sex workers, as well as clients of these programs. Women currently or formerly in the sex industry participated through coalition meetings, and discussion on-site at partner organizations. Coalition participants’ experience with the sex industry ranged from street-based sex work to those trafficked for exploitation; accordingly, they preferred the terminology “women who trade sex for money or survival or those who are sexually exploited or trafficked”, subsequently referred to as “in the game” based on participant suggestion.

Through semi-structured participatory discussion the coalition reviewed: a) local [7] and global [4, 5, 9, 10, 34, 35] data on the prevalence, nature, and health impact of violence against FSWs, b) emergent intervention strategies for FSWs [23, 36], and c) brief GBV interventions and recommendations for general populations [37, 38], with the goal of developing an intervention to address violence for FSWs. Partnership meetings and individual feedback sessions were audio-recorded; for occasional cases of anonymity preference in individual sessions, detailed notes were taken. Recordings were transcribed verbatim, thematically coded in Atlas.ti, and iteratively reviewed. In this brief description of intervention development, we present pivotal quotes from coalition members. The coalition considered targeting violence perpetrators, and ultimately prioritized support for survivors as a necessary first step. Violence-related support, information, and connection with services emerged as key goals. One coalition participant explained, *“it is really powerful just to say ‘we have some things that could help keep girls safe in the game.’”* Another described the value of conveying the message that no one deserves abuse, “*[countering] that whole perception like I can’t be raped because I am a ho kind of thing…”.* A brief intervention approach was selected to maximize feasibility and integration within existing services, and respond to participants’ trajectories. One coalition participant explained, “*Maybe they don’t feel like they have time, or they don’t want to make that phone call[right then]… but you know two weeks later when things shift they have the information… and they can take that action step.”* Extensive discussion informed development of safety card, training materials, recommended safety strategies, implementation and delivery.

### INSPIRE intervention (Fig. 1)

INSPIRE consists of a brief, semi-structured dialogue, reinforced with a safety card, for clinic- or community-based implementation. It blends trauma-informed support, validation, safety promotion, and links to services, consistent with guidelines for the health sector response to violence against women [39], and for addressing violence against sex workers [13]. . While implemented at the individual level, INSPIRE responds to the structural forces that blame FSWs for victimization and thus undermine violence-related disclosure, safety behavior, care seeking and access to justice. **Universal discussion of violence in the context of sexual HIV risk reduction** entails a normalizing statement about violence and intentional discussion of violence-related barriers to HIV prevention, e.g., *“We talk with all clients about violence because it is so common, and fear of abuse can make it hard to negotiate safe sex.”* This is a universal awareness-raising step, rather than a violence screener, due to the high prevalence of violence in this population. It is designed to validate experiences and buffer against self-blame, thus increasing confidence in sexual negotiation, and ultimately reducing sexual risk behavior. It represents an invitation to share fears and experiences related to violence and obtain support. **Trauma-informed harm reduction and safety promotion** entails discussion of violence-related harm reduction and strategies to reduce sexual HIV risk, e.g., “Here are some ways women have told us they try to stay safe…how do you stay safe?” Evidence from FSWs [13, 34–36, 40] and extensive discussions with partners informed safety strategies such as creating a code system, and avoiding situations in which substance use or abuse is likely. Suggested safety behaviors can provide direction for reducing danger, and shift power away from potential abusers, thus decreasing violence and reducing opportunities for unprotected and/or abusive sex. Brief **discussion of local support services** clarifies available violence-related care and normalizes use. A discreet, wallet-sized safety card adapted from best practices for partner violence support [41] summarizes the information and includes contacts for local support services.

**Fig. 1:**
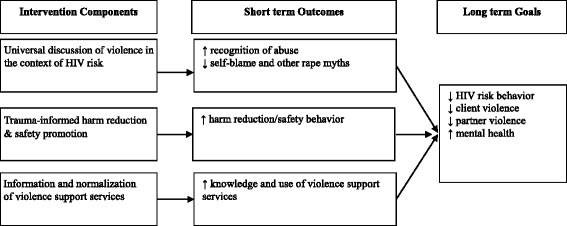
INSPIRE Intervention Model

INSPIRE was implemented by outreach workers intentionally selected for experience with the target population, and interest and experience working with violence survivors. The 4-h intervention training included guidance on handling violence disclosures and extensive practice. The outreach workers met weekly with the research team in the first month to for technical assistance and support; subsequent support was ad-hoc. The field presence of research staff provided additional technical support, particularly during early phases of implementation. INSPIRE’s semi-structured conversation format took an average length of 5–8 min and up to 15 min depending on participant response and needs. For this pilot study, participants received a single dose of INSPIRE. In practice, it is intended for implementation on an ongoing basis, following a low-dose, high-frequency schedule that characterizes FSWs’ relationships with outreach workers.

### Study design

A quasi-experimental, single group pretest-posttest study design evaluated intervention feasibility, acceptability and effect. INSPIRE was implemented in conjunction with the HIV-related mobile van services of the Baltimore City Health Department (BCHD), primarily Needle Exchange Program van, supplemented by the Reproductive Health van. From March to July 2015, recruitment, enrollment and intervention implementation was conducted during all hours of mobile van services at two locations with extensive sex trade activity, one predominantly street-based and the other heavily venue-based. Field research team members were selected based on experience working with the target population, and underwent training specific to sex workers, violence-related research and practice, and ethics in research. Flyers on the BCHD van alerted prospective participants, who approached the research team immediately following care. Eligible participants were ages 18 and over, using BCHD services, and had traded sex for drugs, money, or other resources in the past 3 months. After informed consent, participants completed a self-administered survey (approximately 20 min), with support from the research team in few cases of limited literacy. For this pilot study, all activities were conducted in English language for efficient use of resources and consistency with the demographics of Baltimore city. No participants were turned away for language capacity. Following the intervention, participants completed a brief exit survey, received a gift card, and provided contact information for retention. To support retention, participants provided multiple forms of contact information (phone, email, and names and contact information for up to two proxy individuals to facilitate contact). At follow-up, participants completed a self-administered survey, received resources and a gift card. All were invited to participate in an in-depth interview (25–45 min) with a trained member of the research team. Following a semi-structured guide, interview content focused on sex work context, intervention acceptability, value, and comfort, past experiences being asked about violence if any, changes resulting from the intervention, considerations for using local support services and recommendations for strengthening the intervention.

Intervention implementation and data collection were conducted in private locations, typically on the mobile van or in adjacent vehicles, which deemed feasible and acceptable to participants. Procedures were approved by the Johns Hopkins Bloomberg School of Public Health Institutional Review Board (FWA#0000287), and the Baltimore City Health Department Public Health Review; a federal Certificate of Confidentiality provided additional protection. A waiver of written consent maximized confidentiality.

Of 71 women interested and screened for eligibility, 66 (66/71, 92.96%) were eligible, and 60 (60/66, 90.6%) provided verbal consent and enrolled. Retention at 10–12 week follow-up was 39/60 (65.0%). Public records indicated recent criminal justice involvement and possible incarceration for 6/21 (28.6%) of unreached participants, and one participant was in in-patient drug rehabilitation. At follow-up, all participants were invited to complete an in-depth interview, of whom 17 accepted (17/39, 43.6%); time limitations was the primary reason for non-participation in the interview.

### Measures

Participants provided demographic information including age and race, sex trade contextual information (e.g., age at entry, dependence on income, sex work setting(s), social cohesion with sex workers [42], sex work-related stigma from community and family [43], discrimination) [44], current injection drug use, and sexual risk behavior (e.g., unprotected vaginal sex within the past 30 days, engaging in sexual acts with clients under the influence of drugs or alcohol in the past 3 months).

Client-perpetrated physical or sexual violence was assessed through the Revised Conflict Tactics Scale (CTS) [45] adapted for sex work [10]; two items from the CTS assessed physical or sexual partner violence for participants with partners.

Six items assessed Perception of Abuse in specific situations e.g., client insisting on anal sex after agreement for vaginal sex, with responses on a 4-point Likert scale (possible range 6–24; Cronbach’s alpha = 0.89). Higher scores indicated higher perception of abusiveness.

The 11-item Sex Work-specific Rape Myths Scale was adapted from general-population instruments [46, 47] informed by qualitative work with FSWs [7, 22] and the pervasive myth that sex workers cannot be raped [48]. Items included “Women who trade sex have the right to say no to unwanted sex”, with responses on a 5-point Likert scale (possible range 11–55; Cronbach’s alpha = 0.74). Higher scores indicate greater rape myth endorsement.

The 16-item Sex Work Safety Behavior Scale was adapted from the general-population Safety Promoting Behavior Checklist [49] and tailored for sex workers based on qualitative research, safety recommendations specific to sex workers [13, 34–36, 40], and coalition partners including women currently or recently in the sex industry. Specific safety behaviors are assessed on a 5-point Likert scale (possible range 16–80); higher scores indicate more frequent use.

A 5-item Condom Confidence scale [50] was adapted for sex workers, with responses on a 5-point Likert scale. Due to poor psychometric properties (Cronbach’s alpha = 0.55) and ceiling effects for several items, a single item specific to avoidance of condom negotiation was used, specifically “If I were unsure of a client's feelings about using condoms, I would not ask him to use one”.

Single items assessed participant knowledge and use, respectively, of local support services for intimate partner violence, sexual violence support, and trafficking, respectively, ever (baseline assessment), and since baseline (at follow-up); items specific to sources for assistance with reporting violence to police were assessed only at follow-up.

Depressive symptoms were assessed with the 10-item CESD [51] with responses on a 4-point Likert scale (possible range 0–30; Cronbach’s alpha =0.83). Post-traumatic stress disorder (PTSD) symptoms were assessed with the 17-item PTSD Checklist [52] with responses on a 5-point Likert scale (possible range 17–85; Cronbach’s alpha = 0.96). For both scales, higher scores were indicative of greater symptoms and mean imputation handled small amounts of missing data on specific items.

Intervention acceptability was assessed on the exit survey with a 7-item scale adapted from acceptability measures used in similar studies [53], with responses on a 5-point Likert scale (Cronbach’s alpha =0.96). Items are reported individually for specificity (see Table 1), and responses dichotomized to reflect endorsement (e.g., “agree” or “strongly agree”). A single item assessed participant likelihood of giving the safety card to another individual at risk for violence.

**Table 1:** Participant Demographics and Baseline Characteristics (*n* = 60)

	% (n/n) *(mean,* ±*sd)*
Age *(mean,* ±*sd)*	*(35.5,* ±*7.3)*
Race	
White	72 (41/57)
Black	16 (9/57)
Other	12 (9/57)
Sex Trade Context	
Recruitment site	
Primarily street-based sex work	73 (44/60)
Primarily venue-based sex work	27 (16/60)
Sex work is sole source of income	41 (24/58)
Social cohesion (mean, ±sd)	*(23.60,* ±*9.5)*
Everyday Discrimination (mean, ±sd)	*(28.9,* ±*10.1)*
Sex Work Stigma: Community (mean, ±sd)	*(15.9,* ±*6.0)*
Sex Work Stigma: Family (mean, ±sd)	*(12.9,* ±*5.7)*
Current injection drug use	86 (52/60)
Intervention acceptability	
Likely to give the safety card to someone at risk for violence	90 (54/60)
Helpful to hear about violence support programs	98 (59/60)
Helpful for providers to talk about violence and safety to people like me	98 (59/60)
I would bring a friend here to have this conversation	98 (59/60)
My interventionist cares about my safety	98 (59/60)
I felt comfortable talking with the interventionist	98 (59/60)
I felt safe	98 (59/60)
I felt that what I said would be kept private	98 (59/60)

### Analysis

Descriptive analysis were calculated for baseline demographic characteristics, sex work context, drug use and intervention acceptability parameters. Attrition analysis compared baseline characteristics of those retained with those lost to follow-up via t-test and chi-square analysis. Differences in key outcomes (i.e., attitudes, HIV risk behavior, safety behavior, knowledge and use of support services, violence, and mental health) between baseline and 12 week follow-up were evaluated using paired t-tests and McNemar’s tests, statistical significance was set at *p* < 0.05 and non-significant trends at *p* < 0.10 are also reported for this small pilot study. Sample size varied slightly due to small amounts of missing data. In-depth interview recordings were transcribed verbatim for analysis. An iterative process was used to maximize discovery and allow themes to naturally emerge. Three members of the research team read and open coded an initial set of transcripts to identify initial primary themes; subsequently a set of interviews was dual coded in pairs of independent coders. Major themes identified in the open coding process were refined using axial and selective coding. Remaining interviews were coded by a single investigator with additions to the codebook made by consensus.

## Results

### Baseline characteristics

Average age was 35.3 years, and 72% of participants were White (Table 1). The majority of participants (73%) were recruited from the site where the primary sex work activity was street-based. For 41% of women, sex work was the sole source of income. Most (86%) were current injection drug users.

### Intervention acceptability

Participants reported high intervention acceptability on the exit survey, with approximately 98% feeling comfortable talking with the interventionist, reporting a sense of safety and caring from the interventionist, and that the intervention was helpful. A majority of participants (90%) reported feeling likely to give the safety card to someone else at risk for violence.

### Outcomes analysis

Women’s safety behavior scores increased significantly from baseline to follow-up (51.2 vs. 58.1;*p* < .0001; Table 2). Use of IPV support programs significantly increased from baseline (10.5%) to follow-up (28.9%, *p* < .01). Knowledge of trafficking-related support programs increased from 43.2% to 67.6% (*p* = 0.05), as did use (2.6% to 21.1%, *p* < .01). Knowledge of sexual violence support programs increased from 28.9% to 76.3% (*p* < .0001), as did use from 2.6% to 26.3% (*p* < .01). At follow-up, the majority of women knew at least one program to obtain assistance reporting violence to police (68.4%), and 28.9% reported having used such a program. Endorsement of sex work-specific rape myths decreased between baseline and follow-up but did not reach statistical significance (mean 24.0 vs. 21.9, *p* = 0.11). Avoidance of condom negotiation decreased between baseline and follow-up (2.0 vs.1.4; *p* = 0.04). Average frequency of sex with clients while under the influence of drugs or alcohol decreased significantly (mean = 4.4 vs 4.0; *p* = 0.04). The prevalence of vaginal sex with clients in the past 30 days tended to decrease from 97.4% to 89.5%, *p* = 0.08. Prevalence of recent client physical or sexual violence increased from 28.2% to 43.6% (*p* = 0.03). PTSD and depression symptomatology were high at baseline (PTSD mean = 51.4; CESD mean = 19.2); no changes were observed from baseline to follow-up.

**Table 2:** Intervention endpoints across baseline and follow-up, and attrition analysis

	Full sample*n* = 60	Retained *n* = 39			Lost to follow-up *n* = 21	
	Baseline % *(mean,±sd)*	Baseline % *(mean,±sd)*	Follow-up % *(mean,±sd)*	*p* value^§^	Baseline % *(mean,±sd)* ^§§^	Attrition analysis*p* value^§§^
SHORT TERM OUTCOMES						
Attitudes						
Recognition of abuse (possible range 6–24)	*(9.2, ± 3.9)*	*(9.3, ±4.2)*	*(10.0,± 4.6)*	0.22	*(9.0,± 3.2)*	0.80
Sex work-specific rape myths (possible range 11–55)	*(24.1,± 6.8)*	*(24.0, ± 7.4)*	*(21.9,± 7.6)*	0.11	*(24.2 ± 5.8)*	0.91
Safety behavior						
Sex work safety behavior scale (possible range 16–80)	*(52.5,± 13.8)*	*(51.2,± 13.8)*	*(58.1,± 12.7)*	**<0.001**	*(54.7,± 14.8)*	0.36
Knowledge and use of support services^a^						
Knowledge of intimate partner violence support programs	88.1	92.1	89.5	0.56	81.0	0.21
Use of intimate partner violence support programs	11.9	10.5	28.9	**<.01**	14.3	0.67
Knowledge of trafficking-related support programs	40.7	43.2	67.6	**0.05**	33.3	0.39
Use of trafficking-related support programs	3.4	2.6	21.1	**<.01**	4.8	0.67
Knowledge of sexual violence support programs	32.2	28.9	76.3	**<0.001**	38.1	0.47
Use of sexual violence support programs	3.4	2.6	26.3	**<0.01**	4.8	0.67
Knowledge of programs to help report violence to police**	--	--	68.4	**--**	**--**	--
Use of police reporting assistance support programs**	--	--	28.9	**--**	**--**	--
LONG TERM GOALS						
HIV Risk Behavior						
Avoidance of client condom negotiation (possible range 1–5)	*(2.0,± 1.3)*	*(2.0,± 1.4)*	*(1.4,± 0.8)*	**0.04**	*(2.0,± 1.4)*	0.89
Frequency of sex with clients under the influence of drugs or alcohol (possible range 1–5)	*(4.5,± 0.9)*	*(4.4,± 0.9)*	*(4.0 ± 1.4)*	**0.04**	*(4.7,± 0.6)*	0.18
Any vaginal sex with clients, past 30 days	98.2	97.4	89.5	0.08	100.0	0.48
Any unprotected vaginal sex with clients, past 30 days	34.6	33.3	36.4	0.76	36.8	0.80
Any anal sex with clients, past 30 days	41.4	35.1	32.4	0.76	52.4	0.20
Any unprotected anal sex with clients, past 30 days	15.8	33.3	50.0	0.32	55.6	0.80
Physical and sexual violence						
Client violence, past 3 months	30.0	28.2	43.6	**0.03**	33.3	0.68
Intimate partner violence, past 3 months (*n* = 23 with a partner)	57.1	52.9	47.1	0.65	42.9	0.60
Mental health						
PTSD (possible range 17–85)	*(51.5 ± 20.7)*	*(51.4 ± 19.9)*	*(49.8 ± 20.4)*	0.61	*(51.6 ± 22.8)*	0.97
Depressive symptoms (CESD;possible range 0–30)	*(18.9 ± 7.6*	*(18.9 ± 7.9)*	*(18.4 ± 7.8)*	0.70	*(18.9 ± 7.5)*	0.99

^§^baseline values compared with follow-up values via paired t-test for continuous measures, McNemar’s Test for binary outcomes

^§§^baseline values compared with that of retained sample via two-sample t-test for continuous measures, chi-2 test for binary outcomes

^a^Baseline assessment refers to lifetime knowledge or use; follow-up assessment refers to knowledge or use since the baseline survey

**assessed only at follow-up

boldface indicates statistical significance at *p* < 0.05

### Attrition analysis

Relative to retained participants, those lost to follow-up reported more frequent use of safety behavior (*p* = 0.05; Table 1) and more frequent anal intercourse with clients (*p* = 0.04; data not shown). No significant differences were identified based on age, race, recent violence victimization, sex under the influence, attitudinal measures, discrimination, nor additional outcome measures at *p* < 0.10 (data not shown).

### Qualitative results

Participants described high levels of comfort with the intervention and the intervention team and overwhelmingly appreciated the opportunity to share experiences with a non-judgmental source of support.

You could really talk to [the interventionist]. She was very approachable. She didn't make it seem like...Well, I know what I'm doing is illegal. I know what I'm doing is wrong. I know that drugs, you would never think they took you to that level. She didn't judge. (Participant 28)

Most women identified new knowledge of violence-related support programs as a highly valuable component of the intervention. The supported discussion served as a catalyst for considering change.

… a lot of programs…I wasn't reaching out for them before. It wasn't until I met you guys, eight weeks ago, where I started thinking about a lot of this. A lot of things are like smacking me in my face. (Participant 11)

Another participant, since learning about violence-related support services, notes, “you shouldn’t have to be afraid” (participant 28). She described that it could prompt collective action to increase safety: “Then, if more of us stick together and say, ‘No, this is how it's gonna be,’ then that's going to be the rule.”

Some participants described how the intervention facilitated connection to formal services.

[after the intervention] I remember that I called a couple of them to see if they could get me some help and everything. The one lady, in the [violence support program], she got me hooked up with a [housing place called The Christian's House, which, hopefully, they'll have a bed for me in eight days (Participant 4)

For others, new knowledge of services allowed participants to support friends and colleagues.

I had given one lady a [safety] card. She was being beat up by somebody that she was dating. She said she went to [violence support program] and got help. (Participant 22)

One explained becoming aware of trafficking support services, which prompted her to make a call for a colleague in danger.

I never heard about [support for sex trafficking victims] before, never. They explained that if I knew somebody that...that was, basically I'd know where to go (Participant 26)

Women emphasized the value of a safe space to discuss experiences with violence, and they described the striking lack of violence-related support elsewhere in their lives.

[talking about violence] actually helps. It's helpful, so it's a real big burden off my chest because I've been holding it in so long, and it's been so much pain where I'm constantly having dreams. It's all I think about. When I think I can talk to somebody, I find out that I can't. Only thing they wanted to talk about was drugs. [pauses, crying]. But it feels good to finally get it out, to be able to vent. (Participant 18)

In considering what had changed since the intervention, participants described the benefit of safety reminders.

How to watch out for myself a little bit more. Be more alert on my situations, my surroundings. (Participant 11)

I remember looking at [safety card]. I'm thinking about it. It was something that triggered me when I started feeling uncomfortable with that man. I started feeling like these are little things that are going off of my mind that I should be paying attention to (Participant 3).

Participants also spoke of enhanced confidence and collective action gleaned from open discussion of topics rarely discussed, including coercive barriers to condom use, and safety.

[in reaction to the intervention] I thought, "Yes, I want to talk about that." I like that we're talking about using condoms and how sometimes there can be force if you refuse to use a condom. How that is just so stupid on the man's part. So making more awareness and making a girl more confident about insisting upon is important to me. And giving me some techniques or just some more mental support. When you're in that situation you think, "Mm-hmm. He gave me money. I'm not sure if I should be so insistent," but when I have this support system and I think back to this, and I feel more apt to insist upon it. (Participant 3)

Another participant explained that the intervention invigorated her intentions to “stay connected”.

Yeah. I'll tell you where I'm going, you tell me where, try to keep each other safe that way. I'm actually going to talk to my friend who I came down here with about it when we leave. (Participant 2)

In contrast, one participant noted limited change as a result of the intervention, owing to entrenched economic challenges and addiction.

Things are pretty much the same. I still am out here. This is how I'm surviving. I live in a hotel. It costs me a lot of money, plus my habit. I have to be out here and get what I need, just so I can get by each day. Nothing's really changed. Nothing bad has happened in the time period (Participant 5)

Addiction and the need for self-sufficiency were also felt to limit the safety and support that could be provided by fellow FSWs, as articulated by one participant

As long as they're standing on the corner with you, or if you have money and you're going to get them high, then they're with you. But when it's time for them to get money on their own, or have an opportunity, or get high with somebody else, or whatever, then they're gone (Participant 7)

Challenges with the intervention and recommendations for change included women’s limited time for intervention participation, particularly among women working in clubs where shift start times are strictly enforced. A small handful of women had low reading comprehension and one mentioned that while the discussion with the interventionist was helpful, she could not read the safety card.

## Discussion

This brief, trauma-informed discussion of safety and HIV risk, and provision of violence-related resources, was found feasible and highly acceptable to FSWs. Significant improvements were observed in short-term outcomes, including knowledge and use of violence support resources, and engagement in safety behavior. Current evidence of improved safety behavior is important given the emphasis on safety behaviors in international recommendations for addressing violence against FSWs [13]. While past intervention approaches have included safety planning elements, ours is the first to quantitatively evaluate safety behavior, and demonstrate improvements following a brief intervention. Positioning safety tips and support resources in a dialogue that recognizes trauma, and is overtly non-blaming and non-judgmental was welcomed by participants, and offers a direct counterpoint to the structural forces that undermine power for FSWs and blame them for sexual violence. Qualitative results suggest that this messaging coupled with reminders for safety enabled confidence in resisting sexual risk, consistent with the improvements in condom negotiation and resistance of sex under the influence observed quantitatively. Our study is also the first to describe levels of knowledge and use of local violence support services among FSWs, thus informing international recommendations that call for ensuring access to violence-related support for FSWs [13].

Impact on longer-term goals was mixed, which may reflect the limited follow-up period. The reductions in sex trade under the influence and avoiding condom negotiation suggest a cascade influence on sexual HIV risk behavior. The lack of movement in mental health outcomes may reflect the low-dose approach and extent of trauma and substance use in this population, in addition to the short follow-up period. The increase in client-perpetrated violence victimization is alarming. It may reflect perpetrator responses to women’s use of safety behaviors, which can be perceived as challenging power/control, though this did not emerge in the qualitative interviews. It is also possible that the observed increase reflects enhanced comfort in reporting violent experiences. Longer-term follow-up is needed to understand if women can ultimately increase safety and reduce exposure to violence with sustained support, skills and safety planning. This finding affirms the need for sustained access to support and justice for FSWs. Despite its value in connecting participants with violence-related care and improving safety behavior, INSPIRE’s individual approach is likely insufficient as a primary prevention strategy. Qualitative results suggest that addiction and entrenched economic needs may limit the changes that can be catalyzed. INSPIRE is likely to be most effective in a combination package that also addresses intimate partners and clients, seek to modify norms that sanction use of violence, and address underlying economic, addiction, and structural issues that enable sustained violence and HIV risk.

While administered only once for the purpose of this study, INSPIRE as implemented at scale is intentionally low-dose, high-frequency. It is intentionally designed to leverage the outreach workers who often represent the first access point for FSWs, and their broad reach and ongoing interactions. Harnessing the existing HIV prevention infrastructure maximizes sustainability, impact and reach. Study results demonstrate the value of engaging lay-professionals in imparting supportive, non-judgmental messages of violence support and safety. The participatory process was invaluable for intervention development, refinement of meaningful, feasible goals, and intervention delivery. Although an intervention of longer duration or greater intensity may have been even more potent, INSPIRE balances the tradeoff between comprehensiveness and reach to participants. The time limitations expressed by participants further emphasize the value of this low-dose, brief approach. Results are timely in informing national recommendations [54] to improve HIV outcomes by addressing violence and trauma, particularly for women and girls.

Chief limitations are the small sample size, lack of a control group, limited follow-up duration and attrition. Retention was a challenge though retention rates are comparable with past research with drug-involved FSWs in the US (3-month follow-up 66–69%) [24]. Justice system involvement, possible incarceration, and inpatient drug rehabilitation explained 7/21 (30%) study participants who were lost at follow-up. Attrition was non-differential with respect to participant characteristics and study outcomes, thus it is expected to affect statistical power but not internal validity, and analyses assumed missing data at random. Without a control arm, the extent to which attitudinal, behavioral, and violence-related outcomes could change over the study duration without intervention is unclear, however the relatively short follow-up period limits the likelihood of secular trends in the absence of intervention. The small sample size precluded multivariate analysis and our ability to understand predictors of observed increases in violence. Our street presence and partnerships enabled monitoring for events or shifts that could alternatively explain observed changes; no such events were observed. The intervention was implemented in two key sex work sites within Baltimore City; generalizability to other settings is unclear. Regarding intervention content, we note that addressing safety behavior does not change the individuals responsible for perpetrating violence, nor the environment that perpetuates abuse. INSPIRE is ultimately intended for implementation in combination with additional elements to address these issues; the current analysis allows precision in understanding the contribution of an individual-level approach. INSPIRE’s messaging explicitly addressed structural forces that blame women for their victimization, while enabling support, violence-related safety behaviors and care-seeking.

## Conclusions

This study is a critical step toward scalable interventions that address trauma and its impact on HIV acquisition and care trajectories for FSWs. While study outcomes were limited to behavioral HIV risk, INSPIRE and other trauma-informed approaches may hold value for biomedical HIV prevention and HIV treatment, in that violence can undermine successful uptake, adherence, and success [55–57], and may hold value for injection-related risk as well. The trauma-informed elements of WHO Clinical Guidelines for responding to violence against women [38] were adaptable and valuable in this setting and population, and aligned with policy guidance for HIV prevention for sex workers [13]. Addressing violence in the context of HIV prevention is feasible, acceptable to FSWs, and can improve safety and reduce HIV risk, thus supporting health and human rights for FSWs.
